# Miscellaneous Items

**Published:** 1890-03

**Authors:** 


Idiosyncrasy Regarding Tannin.—Dr. Lange, of Copenhagen, mentions a case in which a patient presented a rare idiosyncrasy regarding tannin locally applied. He was a gentleman about thirty years of age, who was suffering from catarrh of the nose and pharynx, examination revealing nothing but a reddened and injected condition of the mucous membrane. The pharynx was swabbed out with a solution of tannin of the strength of 1 in 15. Immediately afterwards the mucous membrane became greatly swollen, the nostrils entirely occluded, an irritating watery fluid being secreted. The uvula and soft palate were found to have become œdematous, the former lying on the dorsum of the tongue. Ice was given to suck and cold compresses were applied. About an hour later, the patient complained of a certain degree of stupor and of a distressing general itching affecting the whole of the surface of the body, which presented the appearance of general urticaria. The next day this had disappeared and the swelling of the nasal and pharyngeal mucous membrane had greatly diminished. The patient then mentioned that twice before he had suffered from almost exactly the same symptoms after being treated with tannin—once in the form of a powder, and once in that of solution. On one occasion he had been seized with severe lassitude and prostration, his "brain feeling quite empty," and he had noticed that his urine was quite dark on both occasions.—*Lancet*, Jan. 11, 1890.

Proposed Five Years' Course of Medical Study in Great Britain.—The General Medical Council is seriously considering the propriety of compelling a five years' course of medical study as a requisite to registration and practice in Great Britain. The question will be up for discussion at the next meeting in May. The Royal College of Physicians will probably oppose the scheme, for the present, a preliminary vote having been taken by its council which declares that it does not see its way to recommend the adoption of the increa ed term of study.

An Irish editor says he can see no earthly reason why women should not be allowed to become "medical men."

The Health of London in 1889.—Remarkable as has been the continual decline of the death-rate in England and Wales in recent years, the decrease of the rate of mortality in London, with its aggregate population of more than four millions, with constantly increasing density, is still more remarkable. The Registrar-General, in his last annual summary, reported that the death-rate in registration in London in 1888 was 18.5 per 1,000, being "far the lowest death-rate as yet recorded in London," the next lowest being 19.8, 19 9 and 19.6 in the three immediately preceding years 1885–86-87, previously to which the London death-rate had never fallen below 20 per 1,000. The death-rate in 1889, moreover, again fell, and was considerably below the low rate of 1888. The Registrar-General's return for the fifty-second week of 1889, just issued, affords the means of calculating that the mean annual death-rate in London in the fifty-two weeks of last year did not exceed 17.5 per 1,000, was 1 per 1,000 below the rate in 1888. —*Lancet*, Jan. 4, 1890.

Sir William Gull says: "I think that instead of flying to alcohol, as some people do when they are exhausted, they might very well take food, and would be very much better without the alcohol. If I am fatigued with over work, personally, my food is very simple; I eat the raisens instead of drinking the wine. I have had a very large experience in that practice for thirty years. This is my own personal experience, and I believe it is a very good and true experience. I should join issue at once with those who believe that intellectual work can not be so well done without wine or alcohol. I should deny that proposition and hold the very opposite. There is a great deal of injury done to the health by the habitual use of wines in their various kinds, and alcohol in its various shapes, even in so-called moderate quantities. It leads to the degeneration of the tissues; it spoils the health, and it spoils the intellect."—*Laws of Life.*

Dr. Richardson, in his lecture on "Disease and How to Combat It," says that sunlight in the sick-room has a direct influence on minute organic poisons.

Death Under Chloroform.—A patient, aged sixty-five, was admitted to the Queen's Hospital, Birmingham, with a tumor in the abdomen. An exploratory operation was decided on, and the patient assenting, chloroform was administered by one of the house-physicians. Before complete anæsthesia had been obtained, and before any attempt had been made to commence the operation, the patient suddenly gave one or two gasping inspirations and then ceased to breathe, and the heart stopped. Every effort was made to restore him, but without avail. The heart was carefully examined the day before the administration, and was believed to be quite healthy, an opinion confirmed at post-mortem examination, when no morbid condition was found in that organ. There was malignant disease of the stomach.—*British Med. Journal*, Jan 4, 1890.

Treatment for Catarrhal Affections of the Throat.—Dr. G. B. Hope, 34 W. 51st Street, New York, Attending Surgeon of the Metropolitan Throat Hospital, and Professor of Diseases of the Throat, University of Vermont, says: "For a long time I have been employing Horsford's Acid Phosphate as a constitutional treatment for catarrhal affections of the throat. I consider it to be among the very best tonic excitants of the vocal organs, and particularly applicable in relieving the fatigue and huskiness of voice incident to those who pursue a professional career of actor or vocalist, and far preferable to the various forms of wines now so generally recommended for this purpose. I have seen no other allusion to its employment in this direction, which I believe you are perfectly safe in recommending, both from a theoretical and practical point of view."

Saccharin is said to be a powerful antiseptic. A solution of saccharin of a strength of 1 to 500 is an active germicide. A most efficient, and at once inexpensive antiseptic mouth wash can be made by preparing a six per cent. solution of saccharin in water. A teaspoonful of the drug to a pint of water would about make this proportion.

Some time ago it appeared from statistics that the heads of Americans and Englishmen were growing larger. Quite recently (*Med. Record*) Mr. Edward Atkinson has collected statistics which show that the whole American, as well as his head, is growing bigger. It seems that the Yankee and the Southerner are somewhat similar in figure, and they both have comparatively long legs and small waists. In the West, we are told, the waists are proportionately larger and the legs shorter from "climatological and ethnological causes."

Reducing Fat.—Mr. Davies, in his work on "Foods for the Fat," says that two rats, weighing twelve ounces, were placed on an exclusive diet of lean meat and water. They remained healthy in appearance, but steadily lost weight, and in a month's time weighed only eight and three-quarter ounces. They were now placed on a miscellaneous diet, and in a week's time weighed twelve and one-half ounces.

A Weekly Rest Congress was recently held in Paris, under the presidency of M. Leon Say, the object of which was to urge the general observation of one day of rest in the seven, which is regarded by the members of the congress as necessary for the preservation of the health of man as well as of beast.

In Scotland the Inter-Universities Students' Committee recommends the extension of the medical course to five years, with a short preliminary course on medicine and surgery, and compulsory instruction on the eye and ear, and on operative surgery.

A graduate of the Jefferson Medical College was recently refused a license to practice in Minnesota because his studies had not covered "three courses of at least six months each," as required by the laws of that State.

